# Evaluation of actions, barriers, and facilitators to reducing dietary sodium in health care institutions

**DOI:** 10.1002/fsn3.814

**Published:** 2018-09-30

**Authors:** Michael Lacey, Sharon Chandra, Roula Tzianetas, JoAnne Arcand

**Affiliations:** ^1^ Toronto Public Health Toronto Ontario Canada; ^2^ St. Joseph’s Health Centre Toronto Ontario Canada; ^3^ Saint Elizabeth Toronto Ontario Canada; ^4^ Faculty of Health Sciences University of Ontario Institute of Technology Oshawa Ontario Canada

**Keywords:** food service, hospitals, long term care, menus, policy, sodium

## Abstract

**Background:**

Globally, population‐wide sodium reduction strategies have been adopted and implemented to address the adverse health effects of excess dietary sodium. However, in Canada, minimal coordinated action by governments has occurred, including interventions aimed at food service operations in hospitals and long‐term care (LTC) centers. The objective of this study was to investigate actions, attitudes, barriers, and facilitators related to sodium reduction in these institutions.

**Methodology:**

A cross‐sectional survey was administered to food service administrators working in hospitals and LTC facilities in Ontario. Responses from key informants from 27 institutions, representing 9,823 patient/resident beds were included.

**Results:**

Overall, 63.0% of institutions had an established sodium target (900–4,000 mg/day). The reported sodium level on “regular” menus was 2,845 ± 1,025 mg/day. Sixty‐three percent believed it was important to reduce sodium on inpatient/resident menus. Top facilitators reported for sodium reduction included group purchasing organizations identifying lower sodium foods (85.2%), increased availability of pre‐packaged lower sodium products (77.8%), government prioritizing and providing support and resources (74.1%), and improved taste of lower sodium foods (74.1%). Only 37.0% believed that patient/resident satisfaction would decrease with sodium reduction. Sodium reduction practices were variable among food service operations.

**Conclusions:**

These data support the need for consistent and coordinated policies to facilitate sodium reduction in hospitals and long‐term care settings and for multi‐sectorial government, industry, and institutional support to ensure success.

## 1 INTRODUCTION

Population‐wide sodium reduction strategies have become a major public health focus in Canada and worldwide due to the adverse health and economic effects of high sodium intakes. Excess sodium consumption causes hypertension and is a major modifiable risk factor for cardiovascular disease and stroke (Aburto et al., 2013). In Canada, the average daily intake of sodium is almost double the daily adequate intake (AI) of 1,500 mg/day and well above the tolerable upper intake level (UL) of 2,300 mg/day (Health Canada, 2018; Institute of Medicine, 2005). It is estimated that reducing the average daily sodium intake to approximately 1,660 mg/day would decrease the prevalence of hypertension by 30% in Canada and result in a reduced risk of stroke, cardiovascular disease, and mortality rates, with an annual cost savings of $430 million per year (Joffres, Campbell, Manns, & Tu, 2007; World Health Organization (WHO), 2009).

In 2010, the Canadian federal government adopted sodium reduction goals and recommendations, which were developed by a multi‐stakeholder Sodium Working Group (Health Canada, 2010). Proposed interventions targeted the food supply, education, and research. Of interest, one recommendation was “The Working Group recommends that the federal government, together with provincial and territorial governments, to develop consistent sodium guidelines and procurement policies for use by food service operations in publicly‐funded institutions” (Health Canada, 2010). However, to date, no coordinated federal policies, programs, or implementation strategies have been developed to support these recommendations for public settings including hospitals and long‐term care (LTC) facilities. In addition, there are no known plans for coordinated provincial action, a need clearly articulated by the Canadian provinces and territories (Provincial‐Territorial Ministers of Health, 2012). However, recently Health Canada has held consultation meetings and has put increased emphasis on sodium reduction in health care and long‐term care settings (Health Canada, 2017). The hospital setting is of particular relevance given the vulnerable populations served and the lack of standardized nutritional criteria and external monitoring of foods and nutrients provided to patients. One study among Ontario hospitals showed high sodium levels, with 100% of regular menus exceeding the AI and 86% exceeding the UL (Arcand, Steckham, Tzianetas, L'Abbe, & Newton, 2012). Furthermore, despite legislation of nutrition standards in the Ontario Long‐Term Care Home Act, the mean daily sodium level served at one facility was 4,390 mg/day (Wright‐Thompson & Piche, 2011). Not surprisingly, in 2012, Ontario health and policy stakeholders identified food procurement in public settings, like hospitals and long‐term care, above as a priority for action (Provincial‐Territorial Ministers of Health, 2012).

With the lack of coordinated policy and program implementation on sodium reduction in health care and long‐term care environments, little is known about the current landscape and existing practices and strategies in food service environments within hospitals and LTC facilities. Therefore, the purpose of the current study was to assess and describe sodium reduction strategies adopted in health and long‐term care settings and to examine current practices, barriers, facilitators, and attitudes related to sodium and sodium reduction. Such data will inform action to address sodium levels in publicly funded health care institutions.

## 2 MATERIALS AND METHODS

### 2.1 Study design and participants

A cross‐sectional survey among key informants was conducted between March and May 2014 among a convenience sample of supervisors and managers who operate food service departments in hospitals and LTC facilities in Ontario, Canada. Forty questionnaires were administered in person to attendees at the Ontario Hospital Association Nutrition Conference in Toronto in March 2014 (17 surveys deemed complete were obtained). An additional 97 questionnaires were distributed electronically to the national food service managers’ network of dietitians of Canada in April 2014. Only completed surveys from Ontario from those that had not previously completed the survey were included (10 responses were captured). Only one individual per facility completed the survey. Participants were entered in a prize draw for a gift basket. All participants provided written informed consent. Research ethics board approval was obtained from the University of Toronto (Protocol: 29997, March 16, 2014).

### 2.2 Study questionnaire

The questionnaire included 46 questions about participant and facility characteristics, operational procedures, actions, strategies, attitudes, facilitators, and barriers regarding reducing sodium on the regular patient menu. This survey was based on former surveys administered by our group (Arcand et al., 2013; Wong et al., 2013). Nutrition experts including registered dietitians, nutrition researchers, and food service managers reviewed questions for external content validity.

### 2.3 Statistical analysis

Descriptive statistics were conducted. Categorical variables are presented as frequencies and percentages. Continuous variables are described as means and standard deviations. A 5‐point Likert scale was used for many survey questions: for example, 1 = “Not at all Helpful,” 5 = “Extremely Helpful”; or 1 = “Strongly Disagree,” 5 = “Strongly Agree” except for questions about menu‐based strategies (not operational‐based strategies) as it included a “Not Applicable” selection. For the analysis on reported factors that have helped or would help in lowering sodium on the regular inpatient/resident menus and perspectives of sodium reduction, responses were re‐coded into three categories (i.e., response of 1 or 2 “Not Helpful” or “Disagree”; 3 was considered “neutral”; 4 or 5 = “Important” or “Agree”). Responses for reported strategies were re‐coded in the same manner except with an additional fourth category (6 = “Not Applicable,” which was not included when calculating means). Surveys must have had at least 80% of questions answered in order to be considered complete. All statistical analyses were performed using the Statistical Package for Social Sciences (SPSS, version 22, Armonk, NY, USA).

## 3 RESULTS

There were 27 informants who, in totality, oversaw food service operations for 9,823 inpatient/resident beds in Ontario hospitals and LTC institutions. Table 1 presents participant demographics and facility characteristics. The majority of respondents were food service directors, managers, or dietitians (85.2%) from either LTC rehabilitation/chronic care (40.7%) or acute care (40.7%). Approximately half of the facilities (52.4%) provided >1,000 inpatient/resident meals per day, with most utilizing a selective menu (92.6%) and approximately half (51.9%) using a 14–28 day menu cycle.

**Table 1:** Participant demographics and health care facility characteristics (*n* = 27)

	*n* (%)
Job title	
Director/manager/dietitian	23 (85.2)
Supervisor/diet technician/other	4 (14.8)
Education	
Nutrition management diploma	9 (33.3)
Undergraduate degree in nutrition	7 (25.9)
Registered dietitian (undergraduate‐level training)	9 (33.3)
Registered dietitian (master's‐level training)	2 (7.4)
Type of health care facility	
Long‐term care/rehabilitation/chronic care facility	11 (40.7)
Acute care hospital	11 (40.7)
Combination of the above	5 (18.5)
Total estimated number of inpatient/resident meals per day	
0–200	1 (4.8)
201–400	2 (9.5)
401–999	7 (33.3)
>1,000	11 (52.4)
Menu type	
Selective menu	25 (92.6)
Non‐selective menu	2 (7.4)
Length of menu cycle	
7–14days	8 (26.0)
>14–28days	14 (51.9)
>1month	2 (7.4)
Two cycles: 7days and 21days	3 (11.1)

### 3.1 Sodium reduction goals and reported sodium levels

Overall, 92.3% of the facilities had adopted goals or strategies to reduce sodium on the inpatient/resident regular menu, with 63.0% having a set of sodium reduction goals/targets (Table 2). The sodium target levels varied, from 900 to 4,000 mg/day; however, more than half of the facilities (53.9%) had set an overall target that exceeded the UL of 2,300 mg/day. Approximately one‐third of facilities (29.4%) indicated they were not meeting the sodium reduction target set for their facility. In 64.3% of facilities, the sodium target established did not include sodium obtained from salt packages or shakers on patient trays. The most common methods for establishing a sodium target were by setting an average per day (33%) or average over a week (33%). When asked about the actual amount of sodium provided on the regular menu, responses ranged from 900 to 5,000 mg/day. Of the 14 facilities that provided salt shakers or salt packages on the tray 39.1%, the reported estimated amount did not account for sodium derived from those sources.

**Table 2:** Sodium reduction goals and reported sodium levels

Actions	Survey respondents(*n* (%)
Have implemented at least one initiative to reduce sodium on the patient/resident menus	25 (92.3)
Have established a sodium target for the regular inpatient/resident menu	17 (63.0)
Current established daily sodium target (mg/day)^a^	
<1,500 mg/day	1 (7.7)
1,500–2,300 mg/day	5 (38.5)
2,300–3,000 mg/day	5 (38.5)
>3,000 mg/day	2 (15.4)
Sodium targets were established by the following means^a^^,^ b	
Per meal	2 (22.2)
Average per day	3 (33.3)
Average over a week	3 (33.3)
Average over a menu cycle	1 (11.1)
Reported sodium levels of regular menus	
Mean ± *SD* mg/day	2,845 ± 1,025
<1,500 mg/day	1 (5.3)
1,500–2,300 mg/day	4 (21.1)
2,300–3,000 mg/day	9 (47.4)
>3,000 mg/day	5 (26.3)
Provide salt packages or shakers on patient trays/tables	14 (58.3)

*Notes*

Continuous variables presented as mean ± *SD*.

^a^Responses only included participants who reported having an established sodium target for the regular inpatient/resident menu. ^b^This was presented as an open‐ended question in the survey. Responses were collapsed into the following categories. Only nine participants responded to this question.

### 3.2 Barriers and facilitators to sodium reduction

Table 3 summarizes several operational‐level factors, barriers, and facilitators to lowering sodium on the inpatient/resident regular menu. The top reported were: support from group purchasing organizations to identify lower sodium foods (85.2%), increased availability of pre‐packaged lower sodium products (77.8%), government prioritizing and providing support and resources to health care facilities in achieving targets (74.1%), and improved taste profile of lower sodium foods (74.1%). When asked about menu‐based strategies to lower sodium on the inpatient/resident regular menu, the strategies considered most helpful were reformulating recipes (72%), substituting current menu items with lower sodium products (70.4%), and the use of herbs and spices (66.7%). Moreover, all menu‐based strategies in the survey were considered helpful by more than half of respondents (Table 4).

**Table 3:** Operational‐based barriers and facilitators to sodium reduction

	Not helpful *n* (%)	Neither helpful nor unhelpful*n* (%)	Helpful *n* (%)
Support from group purchasing organizations to identify lower sodium foods	2 (7.4)	2 (7.4)	23 (85.2)
Increased availability of pre‐packaged lower sodium products	5 (18.5)	1 (3.7)	21 (77.8)
If the government made it a priority to reduce sodium and provided support to health care facilities in achieving targets	1 (3.7)	6 (22.2)	20 (74.1)
Improved taste profile of lower sodium foods	0 (0.0)	7 (25.9)	20 (74.1)
More time to dedicate to improving the menu and finding lower sodium alternatives	3 (11.1)	7 (25.9)	17 (63.0)
Providing education and awareness to staff and patients about the benefits of lowering sodium in the diet	4 (14.8)	7 (25.9)	16 (59.3)
Increased purchasing of food from local food producers to provide foods that are lower in sodium	7 (25.9)	10 (37.0)	10 (37.0)
Increased food budget	3 (11.5)	8 (30.8)	15 (57.7)
Increased staffing budget	6 (23.1)	7 (26.9)	13 (50.0)
Support from upper management at facility	7 (25.9)	9 (33.3)	11 (40.7)
Change in production/kitchen equipment	14(51.9)	7 (25.9)	6 (22.2)
Decrease in menu cycle length	23(85.2)	3 (11.1)	1 (3.7)

**Table 4:** Menu‐based strategies to sodium reduction

	Not helpful *n* (%)	Neither helpful nor unhelpful*n* (%)	Helpful *n* (%)	Not applicable *n* (%)
Reformulating recipes	3 (12.0)	2 (8.0)	18 (72.0)	2 (8.0)
Substituting current menu items with lower sodium products	1 (3.7)	6 (22.2)	19 (70.4)	1 (3.7)
Use of herbs and spices	2 (7.4)	4 (14.8)	18 (66.7)	3 (11.1)
Removing salt packages from tray	4 (15.4)	0 (0.0)	17 (65.4)	5 (19.2)
Creating sodium limits for individual foods and dishes	1 (3.7)	7 (25.9)	17 (63.0)	2 (7.4)
Preparing more fresh meals/reducing use of prepared foods	4 (14.8)	2(7.4)	15 (55.6)	6 (22.2)

### 3.3 Perceptions of sodium reduction

Overall, 63.0% of respondents believed it was important to reduce sodium on inpatient/resident menus and 53.8% believed that it was important to reduce sodium in retail operations in health care facilities (Table 5). Only 37.0% of respondents believed that patient satisfaction would be decreased if sodium was reduced on inpatient/resident menus, and 40.7% believed that lowering sodium levels on patient menus would improve the overall health of patients.

**Table 5:** Reported perceptions toward dietary sodium reduction in food service supervisors and managers

	Disagree *n* (%)	Neither agree nor disagree)*n* (%)	Agree *n* (%)
I limit the amount of sodium in my personal diet and/or the diet of my family members	0 (0.0)	7 (25.9)	20 (74.1)
I believe it is important to reduce sodium on inpatient/resident menus in health care facilities	0 (0.0)	10 (37.0)	17 (63.0)
I believe it is important to reduce sodium on the menus of retail operations in health care facilities	1 (3.8)	11 (42.2)	14 (53.8)
I have enough guidance and direction regarding how to reduce sodium at my facility	5 (18.5)	8 (29.6)	14 (51.9)
Lowering sodium levels of patient menus will improve the health of the patients in my facility	4 (14.8)	12 (44.4)	11 (40.7)
Patient satisfaction would be decreased if sodium was reduced on inpatient/resident menus	9 (33.3)	8 (29.6)	10 (37.0)
There is so much controversy about sodium that I am uncertain about what level of sodium is most appropriate	15 (55.6)	5 (18.5)	7 (25.9)

## 4 DISCUSSION

This is the first known study that has explored practices, attitudes, barriers, and facilitators toward sodium reduction in hospitals and LTC facilities. A large proportion of facilities studied had established sodium reduction targets for the regular inpatient/resident menu. However, target sodium levels were highly variable across facilities, ranging from 900 to 4,000 mg/day, with more than half of these targets exceeding the UL of 2,300 mg/day. One‐third of facilities reported not meeting their established sodium target, providing a reported average of 2,845 mg/day (range 1,000 to 4,500 mg/day); however, this is likely an underestimate since 42% of facilities did not account for salt added from packages or shakers. This observation is consistent with published literature that demonstrates high sodium levels in LTC facilities (Wright‐Thompson & Piche, 2011) and hospitals (Arcand et al., 2012). Implementation of sodium reduction is clearly challenging in health care settings, while balancing other priorities of food quality, nutritional value, labor cost, and food costs (Dietitians of Canada, 2015).

This study found broad support for coordinated multi‐sectorial action and intervention on sodium reduction, including the government, group purchasing organizations, hospital administration, and within food service departments themselves. Other jurisdictions, such as British Columbia (BC), Alberta, and New York City, have successfully implemented plans to reduce the amount of sodium served in health care settings (Alberta Health Services, 2013; Kimmons, Wood, Villarante, & Lederer, 2012; Ministry of Health, Population and Public Health; Niebylski et al., 2014). Building on best practices and lessons learned from these jurisdictions could help facilitate the execution of a clear‐cut structured regulatory approach in Ontario for setting targets and adopting strategies for sodium reduction. Monitoring of sodium levels may ensure consistent implementation of the adoption of sodium reduction targets. This monitoring should not only occur at the service level but also by third parties, and include assessments of hospital foods served to determine if they are meeting sodium reduction benchmark targets (Health Canada, 2013). Monitoring and evaluation are critical since having target sodium levels for health care institutions or foods along, set forth by legislation or by voluntary measures, may not be sufficient to achieve success (Arcand et al., 2012). This highlights the essentiality of both system‐level changes and action at the institutional level.

In our survey, the top‐rated actions that respondents felt they could employ at an institutional level were reformulating recipes, substituting current menu items with lower sodium products, and the use of herbs and spices to replace salt. Sodium reduction strategies move along a continuum from “easy fixes” (i.e., removal of sodium packages on trays) to “complex fixes” such as more funding for staff to produce food from scratch. There have been no known studies examining the most effective strategies to employ institutionally in hospitals or LTC to address sodium reduction. Our survey respondents reported that limiting the use of high sodium packaged foods and/or high sodium recipes is a key strategy. This includes an increased availability of lower sodium packaged foods. It is important that sodium reduction strategies consider food and labor costs. The current high use of prepared foods in hospitals and LTC facilities may be due to the significant labor costs associated with scratch preparation (Health Canada, 2013; Moulson, 2012). Additionally, in this study, we identified a role for group purchasing organizations, which was to encourage reformulation among food manufacturers and communicate information about new and reformulated products to food service operators. Group purchasing contracts are an opportunity to define specifications for the sodium content of foods procured, triggering reformulation and increasing demand and buying power for lower sodium foods (Dietitians of Canada, 2015). Currently, group purchasing contracts and specifications exist at regional levels; however, broader impact would result with such procurement guidelines if coordination occurred provincially or, ideally, nationally. Finally, the attitudes of individuals responsible for lowering the sodium content on the menu may be associated with the desire to take action to implement steps to reduce sodium. In this study, we found that the majority believe it is important to reduce sodium not only on inpatient/resident menus in health care facilities, but also on the menus of hospital retail operations as well. Importantly, we found that the majority of food service operators felt that sodium reduction would have a neutral effect or would not reduce patient satisfaction with the menu. Additionally, we observed that approximately one‐quarter of respondents were uncertain about the benefits of sodium reduction in health, which may be related to media attention placed on low‐quality controversial studies. This points to ongoing educational efforts that may be required to translate scientific knowledge to this audience to increase confidence in the application of scientific evidence in the health care setting.

There are limitations with this study. The sample size was small and limited to facilities in Ontario; however, those surveyed service a large number of individuals, representing 9,823 inpatient/resident beds in Ontario, and thus represent a relatively large population. The numbers included diverse types of health care centers across Ontario, where there are 145 hospitals in Ontario (Ontario Hospital Association, n.d) and 630 long‐term care homes (Ontario Long Term Care Association, 2016). This study also relied on self‐reporting of sodium levels in regular menus, which may be biased as underestimates; however, the high sodium levels reported underscore a need for action and for the importance of conducting planned prospective monitoring of sodium levels in these environments. These data should inform a larger cross‐sectional analysis that captures data from other provinces and that prospectively calculates sodium levels. The province of Ontario is similar to many other jurisdictions, as it currently has minimal provincial‐level interventions related to dietary sodium in foods and menus served in public institutions.

## 5 CONCLUSION

There is large variation between health care facility approaches and sodium reduction in Ontario, including target levels set, actions taken, and success achieved, which is not surprising since there is no national or provincially led sodium reduction strategy for publicly funded institutions. This study identified food service operator support for a coordinated multi‐sectorial approach on sodium reduction for health care institutions including commitments by federal and provincial governments, food manufacturers, group purchasing organizations, and actions at the individual food service provider level.

## CONFLICT OF INTEREST

The authors declare that they have no conflict of interests.

## AUTHOR CONTRIBUTIONS

M.L. and S.C. were responsible for survey development, validation, data collection, data analysis, data interpretation, and preparation of the first draft of the manuscript. R.T. was assisted with survey development and data interpretation and provided critical review and editing of the final manuscript. J.A. oversaw all aspects of the study and was responsible for study conception and design, survey development and validation, data interpretation, and provided critical review and editing of the final manuscript.

## TRANSPARENCY DECLARATION

The lead author affirms that this manuscript is an honest, accurate, and transparent account of the study being reported. The reporting of this work is compliant with STROBE guidelines. The lead author affirms that no important aspects of the study have been omitted and that any discrepancies from the study as planned have been explained.

## ETHICAL STATEMENT

No authors have conflict of interest to report. This study was approved by the Research Ethics Boards at the University of Toronto and Mount Sinai Hospital, Toronto, Ontario, and conforms with the Declaration of Helsinki. Informed content was obtained by all study participants prior to survey completion.
